# A Comprehensive Quality Evaluation System for Complex Herbal Medicine Using PacBio Sequencing, PCR-Denaturing Gradient Gel Electrophoresis, and Several Chemical Approaches

**DOI:** 10.3389/fpls.2017.01578

**Published:** 2017-09-13

**Authors:** Xiasheng Zheng, Peng Zhang, Baosheng Liao, Jing Li, Xingyun Liu, Yuhua Shi, Jinle Cheng, Zhitian Lai, Jiang Xu, Shilin Chen

**Affiliations:** ^1^Key Laboratory of Beijing for Identification and Safety Evaluation of Chinese Medicine, Institute of Chinese Materia Medica, China Academy of Chinese Medical Sciences Beijing, China; ^2^Key Laboratory of Technologies and Applications of Ultrafine Granular Powder of Herbal Medicine, State Administration of Traditional Chinese Medicine, Zhongshan Zhongzhi Pharmaceutical Group Limited Zhongshan, China; ^3^Guangdong Provincial Key Laboratory of New Drug Development and Research of Chinese Medicine, Guangzhou University of Chinese Medicine Guangzhou, China; ^4^School of Chinese Materia Medica, Beijing University of Chinese Medicine Beijing, China; ^5^Traditional Chinese Medicine Gynecology Laboratory in Lingnan Medical Research Center, Guangzhou University of Chinese Medicine Guangzhou, China

**Keywords:** quality evaluation system, herbal medicine, PacBio sequencing, PCR-DGGE, HPLC, Sanger sequencing, safety issues, Danggui Buxue Formula

## Abstract

Herbal medicine is a major component of complementary and alternative medicine, contributing significantly to the health of many people and communities. Quality control of herbal medicine is crucial to ensure that it is safe and sound for use. Here, we investigated a comprehensive quality evaluation system for a classic herbal medicine, Danggui Buxue Formula, by applying genetic-based and analytical chemistry approaches to authenticate and evaluate the quality of its samples. For authenticity, we successfully applied two novel technologies, third-generation sequencing and PCR-DGGE (denaturing gradient gel electrophoresis), to analyze the ingredient composition of the tested samples. For quality evaluation, we used high performance liquid chromatography assays to determine the content of chemical markers to help estimate the dosage relationship between its two raw materials, plant roots of Huangqi and Danggui. A series of surveys were then conducted against several exogenous contaminations, aiming to further access the efficacy and safety of the samples. In conclusion, the quality evaluation system demonstrated here can potentially address the authenticity, quality, and safety of herbal medicines, thus providing novel insight for enhancing their overall quality control.

**Highlight**: We established a comprehensive quality evaluation system for herbal medicine, by combining two genetic-based approaches third-generation sequencing and DGGE (denaturing gradient gel electrophoresis) with analytical chemistry approaches to achieve the authentication and quality connotation of the samples.

## Introduction

Complementary and alternative treatments for illnesses forms an important part of the modern medical system, which contributes to the healthcare and wellbeing of many human populations. Complementary and alternative medicines are often derived from plant, animal, and mineral materials, among which herbal medicines stand out as the form most widely used (WHO-WIPO-WTO BOOK^1^). However, quality issues concerning herbal medicines such as adulterants from related and unrelated species (Heubl, 2010; Wang et al., 2015), poor quality resulting from improper harvesting times, and exogenetic contaminations, namely heavy metals (Ernst, 2002) and pesticide (Chen et al., 2012) are routinely detected, which has the potential to threaten their clinical safety (Chan, 2003; Sakurai, 2011). Therefore, suitable and reliable evaluating approaches are now urgently needed to safeguard the quality of herbal medicines.

Species identification is crucial for guaranteeing the safety of herbal medicines. Traditional approaches of authentication for herbal medicines are based upon organoleptic characteristics and physical properties (Zhang, 2002), which depend heavily on subjective judgment that often leads to misjudgment (Huxtable, 1990). In the past decades, several chromatographic and spectroscopic methods have been applied to the authentication and evaluation of herbal medicines (Liang et al., 2004), including TLC (thin layer chromatography) (Mangold, 1961), HPLC (Majors, 1972), violet-visual spectrum (VVS), and near infrared spectrum (NIR) (Huck, 2015). However, these approaches remain non-specific, because the chemical profiles of herbal medicines are so similar to closely related plant species and could also be affected by physiological and storage conditions (Dhami and Mishra, 2015). Combining of HPLC with other, more advanced detectors, including mass spectrum (MS) (Horning et al., 1977) and nuclear magnetic resonance (NMR), offers a more accurate and specific solution than the above mentioned “non-specific” ones (Lindon et al., 2000). In recent years, HPLC has been serving as a powerful tool for determining the content of target chemical(s) and identifying the chemical profile of herbal medicines via a fingerprint analysis (Xie et al., 2006), rather than for identifying species *per se*.

At the same time, molecular approaches have become increasingly popular for authenticating herbal medicines and include both PCR-based and sequencing-based methods. The former has techniques, such as random amplified polymorphic DNA (RAPD) (Williams et al., 1990), restriction fragment length polymorphism (RFLP) (Botstein et al., 1980) and their variants [e.g., amplified fragment length polymorphism (AFLP; Han et al., 2007), inter-simple sequence repeat (ISSR; Choi et al., 2011)], quantitative PCR (qPCR) (Holland et al., 1991), loop-mediated isothermal amplification (LAMP) (Notomi et al., 2000; Zhao et al., 2016) and high-revolution melting (HRM) (Wittwer et al., 2003), which are sensitive and fast. The sequencing-based approach involves DNA barcoding (Hebert et al., 2003) and high throughput sequencing (HTS) (Kircher and Kelso, 2010), which when coupled, offer detailed sequence information for authentication. DNA barcoding exhibits great potential for its high inter-specific and intra-specific discrimination ability. However, its application was often previously limited to samples consisting of a single species, and thus precluded complex mixtures such as a medicinal preparations, largely due to the inherent limitations of Sanger sequencing. Fortunately, with the available applicable markers ITS2, *trnL*, and 16s rRNA, HTS has been successfully used to determine the plant composition of herbal preparations at the species level (Coghlan et al., 2012, 2015; Cheng et al., 2014; Hawkins et al., 2015; Ivanova et al., 2016; Raclariu et al., 2017), demonstrating its advantages and reliability in the authentication of complex mixture samples, even if the contaminating species occur at a very low abundance. Compared with short-read sequencing technologies, such as the Ion Torrent (Margulies et al., 2005) and Illumina platforms (Kircher and Kelso, 2010), long-reads sequencing technologies, namely the single-molecule real-time (SMRT) sequencing used by Pacific Bioscience (PacBio), can generate longer reads of several kilobases with a unique circular template; this allows each template to be sequenced multiple times (Eid et al., 2009) to generate a CCS. Because of this, PacBio sequencing can be used for large structural and transcriptomic research projects (Goodwin et al., 2016). It also facilitates the direct sequencing of short DNA templates of metabarcoding.

Polymerase chain reaction-denaturing gradient gel electrophoresis (PCR-DGGE) has served as a powerful tool for detecting gene mutations (Forrest and Cotton, 1990). DNA separation in DGGE is based on the electrophoresis mobility of a partially melted DNA molecule in polyacrylamide gels containing a linearly increasing gradient of denaturants (Fischer and Lerman, 1983). Sequence variation within the melting domains of the DNA fragments causes migration differences in the denaturing gradient. PCR products containing a variety of amplicons from different DNA templates can be separated in DGGE because of differences in the sequence base pairings. PCR-DGGE became a common approach to explore the genetic diversity of complex microbial populations (Muyzer et al., 1993; Fukuda et al., 2016). As of yet, there are no reports of using PCR-DGGE to analyze the species composition of one or more complex herbal medicines.

With respect to the sequencing-based approaches and PCR-DGGE, it is crucial to select one or more proper biomarker(s) for species authentication. Among the candidate biomarkers, the internal transcribed spacer 2 (ITS2) has been validated with thousands of herbal species and since recommended as a standard DNA barcode (Chen et al., 2010). In addition, the chloroplast non-coding region *psbA-trnH* has been demonstrated to be reliable for species authentication of flowering plants (Kress and Erickson, 2007; Fazekas et al., 2008); it, too, was recommended as a complementary barcode to ITS2 (Chen et al., 2010). Coupling ITS2 and *psbA-trnH* regions, a species authentication system for the DNA barcoding of herbal materials was established, containing 78,847 sequences of 23,262 species, which altogether covers 95% of the raw herbs in pharmacopeia of China, United States, Japan, Korea, and India (Chen et al., 2014).

Nevertheless, emphasis has been laid on analyzing the biological composition of herbal medicines, yet little attention has been paid to the quantification of their components, which we believe to be an important yet underappreciated factor in quality control. This is because the dosage of the components is responsible, at least in part, for the pharmacological effects of a given herbal medicine. Further, the presence of exogenous contaminations, including those of heavy metal, pesticide, aflatoxins and microbes, etc., represent common safety issues for herbal medicines. Although invisible, these contaminants always leave a trace amount, which demands a series of detections by high-precision instruments.

In sum, the current quality control used for herbal medicines remains unsatisfactory and must be improved by establishing a thorough and robust quality evaluation system. Only by doing so can we better understand and access the quality connotation of herbal medicines. In this study, we report on a series of quality control experiments on a classic herbal medicine, DBF, including several authentication and quality evaluation approaches, with the aim of exploring a comprehensive quality evaluation system for complex herbal medicines. DBF contains 30 g of *Astragali Radix* (i.e., dried roots of *Astragalus membranaceus*, Chinese name Huangqi, designated as HQ) and 6 g of *Angelicae sinensis Radix* (i.e., dried roots of *Angelica sinensis*, Chinese name Danggui, designated as DG), mixed in a ratio of 5:1 by weight. First recorded in the “*Neiwaishang Bianhuo Lun*,” by Li Dongyuan in AD 1247, DBF has since been used clinically in China for centuries. According to modern clinical reports, DBF is a reliable prescription frequently used for promoting blood regeneration and enhancing immunity (Cui, 2005). Here, we first used the TGS and PCR-DGGE to analyze the ingredient composition of the DBF samples based on the ITS2 and *psbA-trnH* regions, while the Sanger sequencing of cloned-insert served as a validation to the results of TGS. Next, we used HPLC to determine the sample content of the chemical markers to help estimate the actual dosage relationship between the two key herbal components. Finally, a series of surveys were carried out against several exogenetic contaminants, to further evaluate the safety of the DBF samples. All the three parts mention above contributes to the quality control system that we recommend for herbal medicine.

## Materials and Methods

### Sample Collection

Three batches each of HQ and DG were manufactured and provided as ultrafine granular powder by the Zhongshan Zhongzhi Pharmaceutical Group (Zhongshan, China). To ensure their species accuracy, all of the independent samples were identified via DNA barcoding and Sanger sequencing. These samples were then ground into fine powder. The DBF samples were formulated in the laboratory by mixing together HQ (30 g) and DG (6 g) according to the prescription dosage, with three replicates. For the TGS sequencing, a known authentic standard sample of DG served as the positive control; it was independently processed in the same way as the other DBF samples.

### DNA Extraction and PCR Amplification

The DNA of the DBF samples was extracted by using the DNAsecure Plant Kit (Tiangen Biotech, DP320, China). After elution, concentrations of the sample DNA were determined by a Nanodrop 2000 spectrophotometer (Thermo Scientific, United States), and then it was stored at -20°C. Individual PCR amplifications of the ITS2 and *psbA-trnH* regions were carried out in a 100 μL PCR system with the following final concentrations: 1 × *Taq* PCR Mix solution (Aidlab, 271447AX, China), 1 μM of each primer, and ∼100 ng of the sample DNA templates. Tagged primers, used for the preparation of the sequencing library, consisted of universal primers with 14-bp tags attached to the 5′ end. The specially designed tags included 8-bp protected bases and 6-bp group-specific tags corresponding to the specific samples. For ITS2, the universal primers P3 and E4 (see **Supplementary Table S1**) were used with the following cycling conditions: 94°C for 5 min, then 40 cycles of (94°C for 30 s, 56°C for 30 s, 72°C for 45 s), and 72°C for 10 min. For *psbA-trnH*, the primers PA and TH were used with the following PCR conditions: 95°C for 4 min, then 35 cycles of (94°C for 30 s, 55°C for 1 min, 72°C for 1 min), and 72°C for 10 min. Double-distilled water served as the negative control, while the authentic DG sample served as the positive control. Electrophoresis of these PCR products was performed on 1% agarose gel. The positive PCR products were then purified by Agencourt^®^ AMPure^®^ XP beads (Beckman Coulter, 15824300, United States) by 0.8 × volume, then washed by 70% ethanol twice and dissolved by double-distilled water. Concentrations of the purified PCR amplicons were determined by a Nanodrop 2000 spectrophotometer (Thermo Scientific, United States). Before the real PCR amplification, the primers used in this study were submitted to do the *in silico* test (Ye et al., 2012) on NCBI^2^ using default parameters. Based on the thousands of *BLAST* hits, the results (see **Supplementary Table S2**) demonstrated that these primers can be used to amplify the target regions, both ITS2 and *psbA-trnH*, from a good number of plant species.

### PacBio Circular Consensus Sequencing and Data Analysis

**FIGURE 1:**
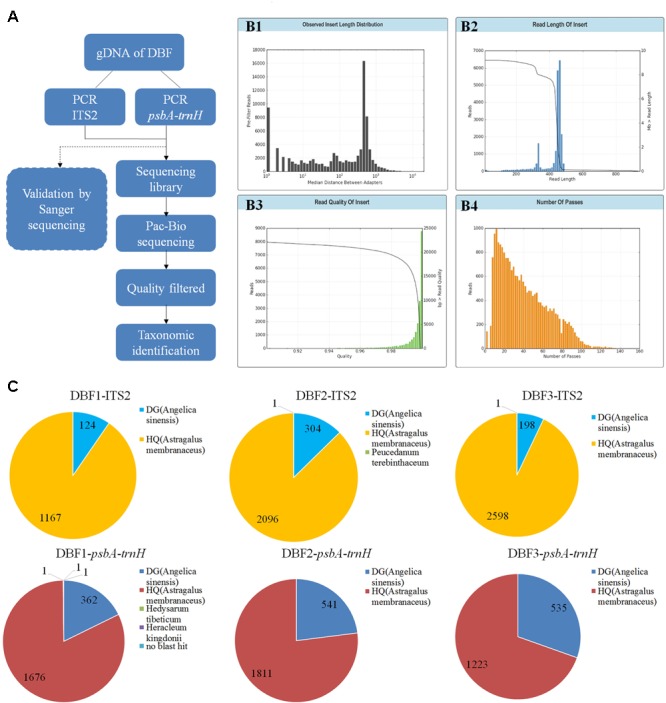
PacBio sequencing procedure and results. The experimental procedure of PacBio sequencing is summarized **(A)**. The read length distribution before filtering **(B1)**. After quality filtering, the sequencing data underwent several quality evaluations to generate another read length distribution **(B2)**, and statistics for the read quality **(B3)**, and the number passes **(B4)**. **(C)** Pie charts show the detailed annotation results for the filtered reads of ITS2 and *psbA*-*trnH* (for three parallel samples).

Purified PCR amplicons of the ITS2 and *psbA-trnH* regions of the DBF samples were mixed by volume to arrive at an equal concentration per sample. The sequencing library was constructed using this mixture with the SMRTbell^TM^ Template Prep Kit 1.0; then bound with V2 primers, by using the DNA/polymerase Binding Kit P6 V2 and P6-DNA polymerase; and transferred to a 96-well PCR plate for the real-time sequencing by using the C4 reagents with a PacBio RS II instrument. The resulting bas.h5 file was used for the subsequent data analysis. We used SMRT Analysis software (v4.0, Pacific Biosciences) to extract the CCS-fastq sequences from the bas.h5 files. Then, the CCS reads with more than 6 (≥7) passes were filtered for the downstream analysis. Resulting reads belonging to different samples were grouped from the filtered CCS reads according to the sample-specific tags in the primer sequences. Next, we used the CodonCode Aligner (v5.1.5.3, CodonCode Corporation) to perform the sequences alignment to yield contigs with these parameters: end-to-end alignments, min. percent identity = 95.0, min. overlap length = 200, min. score = 150. Finally, the contigs and unassembled reads of each sample were used to perform *BLASTn* searches against two databases, the DNA Barcoding System for Identifying Herbal Medicine^3^ and the GenBank nucleotide NR database^4^, by using a max score ≥ 400 and Ident. ≥ 90% (to define *BLAST* results as effective). These two databases were chosen because they contain most of the sequenced Chinese herbs and their adulterants (taxa species of both *Astragalus* and *Angelica* genus with ITS2 and *psbA-trnH* regions recorded in these two databases were listed in **Supplementary Table S3**). The PacBio CCS procedure was shown in **Figure 1A**.

### Sanger Sequencing of Cloned Inserts

We performed a validation for the PacBio sequencing via the Sanger sequencing of cloned inserts. The vector pMD 19-T (Takara, 6013, China) was used to perform the T-cloning by ligating to the purified PCR amplicons, according to the manufacturer’s manual. Subsequently, the whole reaction system was transferred into a competent cell of *Escherichia coli* (TransGen, CD201-02, China), while an empty vector was transferred as the negative control. The transgenic cell culture was spread onto a LB solid culture plate containing 50 ng/L Ampicillin, on the surface of which 40 μL 2% X-gal and 7 μL 20% IPTG had been spread previously. After overnight cultivation, independent white colonies were randomly selected from every plate to perform the PCR validation with the normal primers. Positive clones were grown in liquid culture and then sequenced with universal primers by the Sanger method.

### PCR-DGGE Assay

The ITS2 fragments of the DBF samples were amplified using two universal primers, S2F and S3R, with a 40 bp GC clamp attached to the 5′ end of the forward primer (see **Supplementary Table S1**). HQ and DG again served as the positive control for this method. The PCR system and amplifying procedure were consistent with that already described. The ensuring PCR products were directly subjected to a DGGE assay following the procedure described by Muyzer et al. (1993). We used a polyacrylamide gel of 6% concentration, which contained a 40–60% denaturing gradient. (The 100% denaturant solution contained 40% deionized formamide and 7 mol/L of urea.). Electrophoresis was performed on the Dcode universal mutation detection system (Bio-Rad, Milan, Italy) in 1×TAE at 60°C, 80 V, for 12 h. After the electrophoresis, the gel was stained with EB (Biotium, Fremont, United States) for 20 min, and viewed by UV transillumination. To further confirm the DNA sequence information of the independent bands on the gel, a PCR amplification with the normal ITS2 primers (see **Supplementary Table S1**) was carried out using a small piece of each band’s gel as the template. Then, a taxonomic identification was performed using *BLASTn* (as described above). The PCR-DGGE procedure was shown in **Figure 3A**.

### HPLC Analysis

For the DBF samples, 0.2 g was extracted in 20 × volumes of 70% methanol by supersonic bath for 30 min, with HQ and DG served as the positive controls. These extractions were then filtered through 0.45 μm thick organic membranes and directly submitted to the HPLC analysis. To do this, we used Agilent equipment (Agilent, 1200 series, United States), with a C_18_ column (Agilent, TC-C18, 250 mm × 4.6 mm, 5 μm) as the solid phase. The mobile phase contained 0.2% formic acid aqueous solution (A) and chromatographic pure acetonitrile (B), and following this procedure: 0–15 min, B: 5–45%; 15–60 min, B: 45–95%. An ultraviolet detector measured the ultraviolet absorption of the elution at the wavelengths of 254 and 316 nm, against HQ and DG, respectively. Authentic standard compounds, FA and C-7-G, were dissolved in 70% methanol and pure methanol, respectively, and diluted to a concentration of 10 μg/mL. For each sample solution and standard solution, 10 μL was injected for the HPLC assay under the conditions already described above.

### Contamination Supervision

The determination of contamination residues was carried out according to the 2015 version of the Chinese Pharmacopeia. Specially, sulfur dioxide residue was measured by the acid–base titration method (Cat.2331 in Chapter 4). Heavy metal residue was detected by the method of inductively coupled plasma mass spectrometry (Cat.2321). Pesticide residue was determined by the gas chromatography method (Cat.2341), while for aflatoxins residue the HPLC method was used (Cat.2351). Microbial limit was calculated by the cultivation method (Cat.1105 and 1106).

## Results

### Ingredient Species Authentication of DBF

Here, we explored two novel molecular approaches to audit biological composition of the samples. However, the two approaches differed in their potential for identifying unknown ingredients in the herbal medicines.

#### PacBio CCS Analysis

At first, a total of 22,374 reads were obtained from the DNA library of DBF. The number was narrowed down to 21,426 reads after quality control, CCS processing, and gene length filtration (to 201–800 bp) (see **B1–B4** in **Figure 1**. The sequencing data was deposited on the Sequence Read Archive (SRA) at NCBI with an accession project number of PRJNA401343). Then, based on the group-specific tags introduced during the PCR amplification, the result reads for each of the six independent individual samples were grouped, yielding in 12,640 CCS reads (data generated by the three abovementioned steps are listed in **Table 1**) to taxonomic identification by *BLASTn*. All these resulting reads, for which the effective rate reached almost 100%, were successfully annotated and the identification of the parallel samples remained consistent. Of the 6,489 CCS reads (amounting to 3,026,050 nucleotides) for the ITS2 fragments, there were 5,861 and 626 reads identified as HQ and DG, respectively. Likewise, of the 6,151 CCS reads (2,607,094 nucleotides) for *psbA-trnH* amplicons, 4,710 and 1,438 reads were identified as HQ and DG, respectively (**Figure 1C** and **Table 2**). The positive control showed the resulting reads of only DG (264 and 25 reads for ITS2 and *psbA-trnH*, respectively); whereas the negative control revealed neither electrophoretic band, nor sequencing library achieved. Further, a few unrelated species were detected in both biomarkers of the DBF samples, including two species of Leguminosae, two of Umbelliferae, and one of unknown (see **Table 3**). This result indicated that the PacBio CCS is sensitive enough to determine contaminated species that generally occur only at a very low abundance. These species were revealed by only one read each; that they occurred randomly in parallel sample indicates no biological contamination during the experimental procedures.

**Table 1:** Sequencing results and statistics of the three-step data processing.

Job metric	Original results	After CCS (201–800 bp)	Further extracted by gene tags
Number of resulting reads	22,374	21,426	12,640
Total sample bases	9,348,991	9,180,147	5,633,144
Average read length (bp)	417	428	446

**Table 2:** Results of the CCS and cluster analysis of the DBF samples with two biomarkers.

	ITS2			*psbA-trnH*			Total
	DBF1	DBF2	DBF3	DBF1	DBF2	DBF3	
CCS reads	1,291	2,401	2,797	2,041	2,352	1,758	12,640
Total sample bases (bp)	601,572	1,120,086	1,304,392	880,601	994,258	732,235	5,633,144
Average read length (bp)	466	467	466	431	423	417	446

**Table 3:** Detected contaminated species in the DBF samples.

Biomarker	Sample	Number of reads	Umbelliferae	Leguminosae		Leguminosae	Umbelliferae	Leguminosae	Umbelliferae	No blast hit
						
			DG (*Angelica sinensis*)	HQ (*Astragalus membranaceus*)	Number of contaminants reads	*Melilotus officinalis*	*Peucedanum terebinthaceum*	*Hedysarum tibeticum*	*Heracleum kingdoni*	
ITS2	DBF1	1,291	124	1,167	0	–	–	–	–	–
	DBF2	2,401	304	2,096	1	–	1	–	–	–
	DBF3	2,797	198	2,598	1	1	–	–	–	–
	**Sum**	**6,489**	**626**	**5,861**	**2**	–	–	–	–	–
*psbA-trnH*	DBF1	2,041	362	1,676	3	–	–	1	1	1
	DBF2	2,352	541	1,811	0	–	–	–	–	–
	DBF3	1,758	535	1,223	0	–	–	–	–	–
	**Sum**	**6,151**	**1,438**	**4,710**	**3**	–	–	–	–	–

#### Sanger Sequencing of Cloned Inserts

**FIGURE 2:**
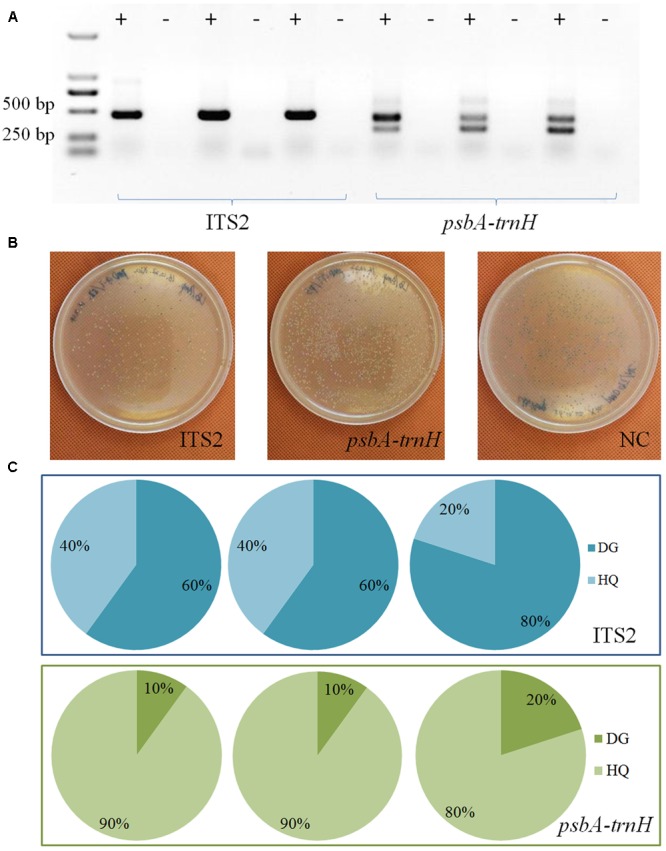
Sanger sequencing of clone insert results. **(A)** The electrophoresis results of the PCR products. **(B)** The colonies on the plates. **(C)** The taxonomic identification results of the colonies by Sanger sequencing for the two biomarkers.

T-A cloning and Sanger sequencing were performed to further validate the authentication result of TGS sequencing. Results showed that all sequences could be identified at the species level (**Figure 2**). A total of 26 and 4 ITS2 sequences were identified as HQ and DG, accounting for 86.7% and 13.3% of all positive sequences, respectively. Meanwhile, 20 and 10 *psbA-trnH* sequences were identified as HQ and DG, accounting for 66.7% and 33.3% out of all sequences, respectively.

Results of TGS sequencing and those of Sanger sequencing were very consistent, in that both HQ and DG were identified with the ITS2 and *psbA-trnH* regions. Nonetheless, the positively identified sequences of both sequencing methods gave different ratios between the two key ingredient species. This discrepancy points to a possible methodological preference in the DNA extraction and PCR amplification, which may cause difficulties in the quantitative measurement of different species’ relative abundances.

#### PCR-DGGE Analysis

**FIGURE 3:**
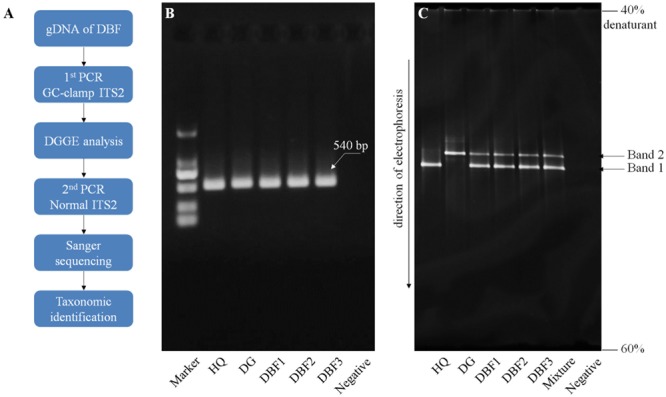
PCR-DGGE procedure and electrophoresis results. **(A)** The PCR-DGGE experimental procedure. Electrophoresis of GC-clamp PCR products are presented in panel **(B)** for the agarose gel and panel **(C)** for the denaturing gradient gel. An equal volume of PCR products from HQ and DG were pooled together to form the “mixture” sample in panel **(B)**.

Internal transcribed spacer 2 fragments with a 40-bp GC clamp were successfully amplified from the three DBF samples and each ingredient sample. In the agarose electrophoresis, the GC-clamp ITS2 fragments of all samples had a similar migration due to the same gene size of c. 540 bp (**Figure 3B**). Interestingly, in the denaturing gradient gel, these amplicons of different species in the mixed sample exhibited a different migration behavior (**Figure 3C**). Two separate bands were observed in the DBF samples, which showed a similar migration pattern to that of their original species as positive controls. To further confirm the DNA sequences of those separated bands, a PCR amplification with the normal ITS2 primers was done by using a small piece of each band’s gel as a template. The Sanger sequencing showed that Band 1 and Band 2 belonged to the ITS2 region of HQ and DG, respectively. Therefore, the PCR-DGGE offered a more visual and rapid ingredient analysis method for DBF than did either of the sequencing methods mentioned above.

### Quality Evaluation of DBF

#### Determination of Chemical Markers by HPLC

**FIGURE 4:**
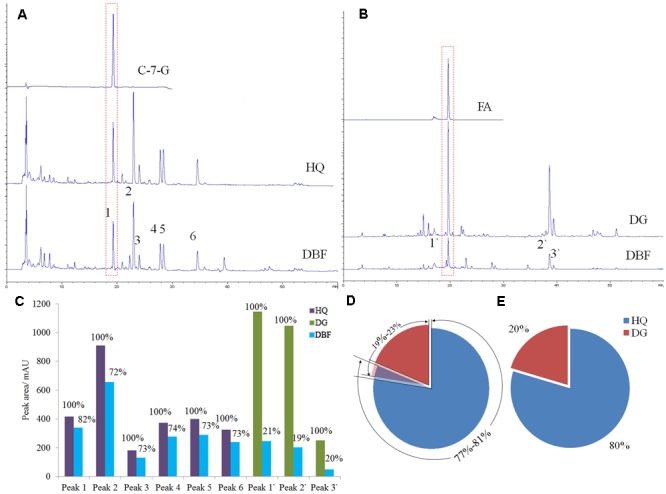
HPLC analysis results of DBF, HQ, and DG. **(A,B)** The absorbent curves of the chemical markers, the independent samples (HQ and DG), and the complex herbal medicine (DBF), observed at wavelength of 254 and 316 nm, respectively. **(C)** The peak areas compared in a bar graph. **(D,E)** The portion of HQ and DG based, respectively, on their common peaks and two chemical markers.

Under the same gradient elution condition, DBF exhibited a similar absorbent curve to HQ at 254 nm (**Figure 4A**), while it was similar to DG at 316 nm (**Figure 4B**). Peak 1 and peak 1′ were identified as the chemical markers C-7-G and FA, respectively, by comparing their retention time with that of authentic standards. The content of C-7-G in DBF was 0.0168%, but reached up to 0.0201% in HQ, whereas the FA content was 0. 149% and 0.0892% in DBF and DG, respectively (as calculated by the standard curve method). To investigate the ingredient content of raw materials, we used the peak areas of common peaks found between HQ and DBF, and DG and DBF, to calculate the relative content of the two main ingredients. Based on the multiple common peaks (**Figure 4C**), the results indicated that HQ accounted for 72–82% of the mixture, whereas DG made up 19–23% of it (**Figure 4D**). At the same time, when considering the chemical markers only, the ratio of HQ to DG content was 4:1 (**Figure 4E**), which was very close to the true value (5:1). The HPLC assay demonstrated that not only is it sensitive at determining the chemical marker(s), but it also provides feasibility to access a reasonable range for the ingredient dosage measurement of herbal medicines which contain raw materials.

#### Determination of DBF Contaminations

Quality control of contaminants includes those of sulfur dioxide, heavy metal, pesticide, aflatoxins, and harmful microbes. The maximum allowable amounts of these contaminants should comply with the *Green Standards of Medicinal Plants and Preparations for Foreign Trade and Economy* and are adjusted according to the latest version of Chinese Pharmacopeia. For the three batch samples of HQ and DG, their contamination residues (see **Supplementary Table S4** for results) were all below allowable limit, thus indicating these samples were safe for clinical use.

## Discussion

With the increasing popularity and demand for herbal medicines, much emphasis has fallen on their safety and quality control. Molecular identification contributes to the biological analysis of complex herbal medicines, thus forming an essential part of “herbal genomics,” which may deepen our understanding of the quality of herbal medicines (Chen et al., 2015). For investigating genetic diversity, Sanger sequencing is a tested and common approach to use (Chen et al., 2015). Yet, it turns out to be quite labor-intensive, time-consuming, and expensive when applied to the species identification of complex herbal medicines. Obviously the situation worsens as the number of ingredients in the herbal medicine increases. By contrast, HTS technology provides a much greater coverage of more samples in a shorter time period, and has better accuracy and repeatability (Jia et al., 2017). Further, introducing indexed tags enables the sequencing of multiple samples in parallel, thus reducing the analyzing time and cost of HTS (Parameswaran et al., 2007). The platform used in the present study, PacBio RS II, is capable of generating long reads and producing a greater sequencing throughput (Goodwin et al., 2016). Yet, a characteristic shortcoming of PacBio is the unsatisfactory sequencing error rate for single-pass sequencing (Schadt et al., 2010; Carneiro et al., 2012). This shortcoming, however, could be overcome by a sequencing strategy of multiple readings on the template DNA. Moreover, the high coverage allows for the differentiation of errors and SNPs (Goodwin et al., 2016). Building on this, we tested PCR-DGGE, a common tool for exploring the genetic diversity of complex microbial populations, to ascertain the species composition of a complex herbal medicine (DBF). Despite encouraging results, the feasibility of this method must be validated through more experiments. Between these two genetic approaches, the PCR-DGGE is suitable for typical labs, while PacBio CCS might be practical in institutions with extensive infrastructure, because it incurs a high cost in both equipment and sequencing, along with a rather sophisticated bioinformatics analyses.

A variety of approaches were developed for matching a query sequence against a reference database. These approaches are classified into four categories, including (1) tree-based methods, e.g., neighbor joining, maximum likelihood, *ATIM* and *SAP*; (2) similarity-based methods, e.g., nearest-neighboring (*NN*), *BLAST, TaxI, BRONX* and *jMOTU*; (3) character-based diagnostics, e.g., *BLOG* and *DNA*-*BAR*; and (4) statistical methods. Several studies were found reporting the comparison of these different analysis methods (Ross et al., 2008; Austerlitz et al., 2009; Virgilio et al., 2010; van Velzen et al., 2012). Yet, the results demonstrated that none of these methods can consistently outperform the others for all data sets (Fan et al., 2014). The other methods, including *BLOG, DNA*-*BAR*, and *NN*, did not outperform significantly greater in identification success rate than *BLAST* (van Velzen et al., 2012). Further, most of the methods have difficulty in coping with large data sets except for *BLAST* (Fan et al., 2014). At the same time, both data bases used in our study are large data sets. The DNA barcoding system for identifying herbal medicine contained 78,847 sequences of 23,262 species, covering 95% of the raw herbs in pharmacopeia of China, United States, Japan, Korea, and India. Not to mention the larger one of GenBank non-redundant nucleotide database. Based on the above, we chose *BLAST* for species identification instead of other methods.

In this study, several contaminated species were detected for the commercial samples, while that no contaminants were found for the positive control sample. This indicated that those contaminated species were possibly introduced during the manufacturing process, rather than the experimental analyzing process. Those contaminated species, which share the same taxa family with both ingredients of DBF samples, are not commonly used medicinal herbs. Therefore, possible explanation for these detected contamination can be misidentification of raw material or unintentional introduction of pollutants during collection of raw material. Moreover, those contaminated species do not exhibit known toxicity, side effect and/or negative interaction, thus raising no safety risks to the usage of DBF samples in clinic. Nevertheless, monitoring the biological composition of herbal medicine is crucial for its safety.

Proposals to combine genetic approaches for species identification and chemical approaches for compound(s) determination have been put forth to better assess the quality of traditional medicines (Calixto, 2000). Quality evaluation of herbal medicines are based on analytical chemistry approaches for determining target constituents or chemical markers (Liang et al., 2009; Li et al., 2011). But it should be noted that safety issues such as contamination from endogenous or exogenous sources, are matter of concern ahead of the efficacy issues. In this study, we investigated a comprehensive quality evaluation system for herbal medicines, one that is concerned with not only robust species identification and chemical analysis, but also the oversight of several potential safety hazards. We hope that this study stimulates new thinking in how to better evaluate the quality of herbal medicines, which should help guarantee their safe and effective clinical use.

## Author Contributions

XZ and PZ contributed to all the researches activities associated with this work. XZ was responsible for writing the manuscript. JL and XL carried out the microscopic and HPLC assays, and also analyzed the data. BL and YS helped with the data analysis of the PacBio and Sanger sequencing. JC and ZL provided the herbal samples and were responsible for determining the extent of contamination in them. JX and SC conceived the study, participated in its design and coordination, and helped to draft the manuscript. All authors read and approved the final manuscript.

## Conflict of Interest Statement

The authors declare that the research was conducted in the absence of any commercial or financial relationships that could be construed as a potential conflict of interest.

## Footnotes

1 https://www.wto.org/english/tratop_e/trips_e/trilatweb_e/ch2d_trilat_web_13_e.htm

2 https://www.ncbi.nlm.nih.gov/tools/primer-blast/

3 http://www.tcmbarcode.cn/en/

4 https://blast.ncbi.nlm.nih.gov

## Supplementary Material

TABLE S1 Primers used in this study.

TABLE S2 Results of primers specificity testing via *in silico* approach.

TABLE S3 Taxa species of both *Astragalus* and *Angelica* genus with ITS2 and *psbA-trnH* regions recorded in the two databases used in this study.

TABLE S4 Determination results of contaminants.

## Abbreviations

C-7-G: calycosin-7-glucoside
CCS: circular consensus sequencing
DBF: Danggui Buxue Formula
DG: Danggui
DGGE: denaturing gradient gel electrophoresis
FA: ferulic acid
HPLC: high performance liquid chromatography
HQ: Huangqi
PacBio: Pacific Biosciences
PCR: polymerase chain reaction
TGS: third-generation sequencing
