# Supplementary material for: A Comprehensive Quality Evaluation System for Complex Herbal Medicine Using PacBio Sequencing, PCR-Denaturing Gradient Gel Electrophoresis, and Several Chemical Approaches
Source: Front Plant Sci. 2017 Sep 13;8:1578. doi: 10.3389/fpls.2017.01578 (PMC5601397; doi:10.3389/fpls.2017.01578)
Supplement: TABLE S1 — Primers used in this study. [file Table_1.doc]

Supplementary Table S1. Primers used in this study

| Direction | Primer | Sequence (5` end to 3` end) | Length (bp) | Applied in |
| --- | --- | --- | --- | --- |
| Forwards | P3-1 | *ATCATCAG***TCGACA**YGACTCTCGGCAACGGATA^*^ | 33 | Third-generation sequencing and Sanger sequencing |
| Reverse | E4-A | *ATCATCAG***TCGACA**RGTTTCTTTTCCTCCGCTTA | 34 |
| Forwards | P3-2 | *ATCATCAG***TATCAC**YGACTCTCGGCAACGGATA | 33 |
| Reverse | E4-B | *ATCATCAG***TATCAC**RGTTTCTTTTCCTCCGCTTA | 34 |
| Forwards | P3-3 | *ATCATCAG***ACGCTA**YGACTCTCGGCAACGGATA | 33 |
| Reverse | E4-C | *ATCATCAG***ACGCTA**RGTTTCTTTTCCTCCGCTTA | 34 |
| Forwards | PA-1 | *ATCATCAG***TATCAC**GTTATGCATGAACGTAATGCTC | 36 |
| Reverse | TH-A | *ATCATCAG***TATCAC**CGCGCATGGTGGATTCACAATCC | 37 |
| Forwards | PA-2 | *ATCATCAG***TGCGTA**GTTATGCATGAACGTAATGCTC | 36 |
| Reverse | TH-B | *ATCATCAG***TGCGTA**CGCGCATGGTGGATTCACAATCC | 37 |
| Forwards | PA-3 | *ATCATCAG***ACGCTA**GTTATGCATGAACGTAATGCTC | 36 |
| Reverse | TH-C | *ATCATCAG***ACGCTA**CGCGCATGGTGGATTCACAATCC | 37 |
| Forwards | GC-S2F | CGCCCGCCGCGCGCGGCGGGCGGGGCGGGGGCACGGGGGGATGCGATACTTGGTGTGAAT | 60 | PCR-DGGE |
| Forwards | S2F | ATGCGATACTTGGTGTGAAT | 20 |
| Reverse | S3R | GACGCTTCTCCAGACTACAAT | 21 |

*: 14-bp special-designed tags in gray background, which is comprised by 8-bp of protective bases (in italic) and 6-bp of recognition bases (in bold).
