# Supplementary material for: A Comprehensive Quality Evaluation System for Complex Herbal Medicine Using PacBio Sequencing, PCR-Denaturing Gradient Gel Electrophoresis, and Several Chemical Approaches
Source: Front Plant Sci. 2017 Sep 13;8:1578. doi: 10.3389/fpls.2017.01578 (PMC5601397; doi:10.3389/fpls.2017.01578)
Supplement: TABLE S2 — Results of primers specificity testing via in silico approach. [file Table_2.doc]

Supplementary Table S2. Results of primers specificity testing via *in silico* approach.

[**Detailed primer reports**](https://www.ncbi.nlm.nih.gov/tools/primer-blast/primertool.cgi?ctg_time=1500460709&job_key=JC77acgrxYPivcC4zdjkirfD9bia0O6lmw)

Primer pair 1

|  | | **Sequence (5'->3')** | **Length** | | **Tm** | | **GC%** | **Self complementarity** | **Self 3' complementarity** |
| --- | --- | --- | --- | --- | --- | --- | --- | --- | --- |
| **Forward primer(P3)** | | GACTCTCGGCAACGGATA | 18 | | 56.14 | | 55.56 | 3.00 | 2.00 |
| **Reverse primer(E4)** | | GTTTCTTTTCCTCCGCTTA | 19 | | 52.88 | | 42.11 | 2.00 | 2.00 |
|  | Number of Blast hits analyzed | | | 55361 | |  | | | |
|  | Entrez query | | |  | |  | | | |
|  | Min total mismatches | | | 2 | |  | | | |
|  | Min 3' end mismatches | | | 2 | |  | | | |
|  | Defined 3' end region length | | | 5 | |  | | | |
|  | Mismatch threshold to ignore targets | | | 6 | |  | | | |
|  | Max target size | | | 4000 | |  | | | |
|  | Max number of Blast target sequences | | | 50000 | |  | | | |
|  | Blast E value | | | 30000 | |  | | | |
|  | Blast word size | | | 7 | |  | | | |
|  | Max candidate primer pairs | | | 500 | |  | | | |
|  | Min PCR product size | | | 57 | |  | | | |
|  | Max PCR product size | | | 1000 | |  | | | |
|  | Min Primer size | | | 15 | |  | | | |
|  | Opt Primer size | | | 20 | |  | | | |
|  | Max Primer size | | | 25 | |  | | | |
|  | Min Tm | | | 57 | |  | | | |
|  | Opt Tm | | | 60 | |  | | | |
|  | Max Tm | | | 63 | |  | | | |
|  | Max Tm difference | | | 3 | |  | | | |
|  | Repeat filter | | | AUTO | |  | | | |
|  | Low complexity filter | | | Yes | |  | | | |

**Primer pair 2**

|  | | **Sequence (5'->3')** | **Length** | | **Tm** | | **GC%** | **Self complementarity** | **Self 3' complementarity** |
| --- | --- | --- | --- | --- | --- | --- | --- | --- | --- |
| **Forward primer(PA)** | | GTTATGCATGAACGTAATGCTC | 22 | | 56.23 | | 40.91 | 8.00 | 3.00 |
| **Reverse primer(TH)** | | CGCGCATGGTGGATTCACAATCC | 23 | | 64.97 | | 56.52 | 8.00 | 8.00 |
|  | Number of Blast hits analyzed | | | 30142 | |  | | | |
|  | Entrez query | | |  | |  | | | |
|  | Min total mismatches | | | 2 | |  | | | |
|  | Min 3' end mismatches | | | 2 | |  | | | |
|  | Defined 3' end region length | | | 5 | |  | | | |
|  | Mismatch threshold to ignore targets | | | 6 | |  | | | |
|  | Max target size | | | 4000 | |  | | | |
|  | Max number of Blast target sequences | | | 50000 | |  | | | |
|  | Blast E value | | | 30000 | |  | | | |
|  | Blast word size | | | 7 | |  | | | |
|  | Max candidate primer pairs | | | 500 | |  | | | |
|  | Min PCR product size | | | 65 | |  | | | |
|  | Max PCR product size | | | 1000 | |  | | | |
|  | Min Primer size | | | 15 | |  | | | |
|  | Opt Primer size | | | 20 | |  | | | |
|  | Max Primer size | | | 25 | |  | | | |
|  | Min Tm | | | 57 | |  | | | |
|  | Opt Tm | | | 60 | |  | | | |
|  | Max Tm | | | 63 | |  | | | |
|  | Max Tm difference | | | 3 | |  | | | |
|  | Repeat filter | | | AUTO | |  | | | |
|  | Low complexity filter | | | Yes | |  | | | |

**Primer pair 3**

|  | | **Sequence (5'->3')** | **Length** | | **Tm** | | **GC%** | **Self complementarity** | **Self 3' complementarity** |
| --- | --- | --- | --- | --- | --- | --- | --- | --- | --- |
| **Forward primer(S2F)** | | ATGCGATACTTGGTGTGAAT | 20 | | 55.13 | | 40.00 | 2.00 | 2.00 |
| **Reverse primer(S3R)** | | GACGCTTCTCCAGACTACAAT | 21 | | 57.49 | | 47.62 | 4.00 | 2.00 |
|  | Number of Blast hits analyzed | | | 52765 | |  | | | |
|  | Entrez query | | |  | |  | | | |
|  | Min total mismatches | | | 2 | |  | | | |
|  | Min 3' end mismatches | | | 2 | |  | | | |
|  | Defined 3' end region length | | | 5 | |  | | | |
|  | Mismatch threshold to ignore targets | | | 6 | |  | | | |
|  | Max target size | | | 4000 | |  | | | |
|  | Max number of Blast target sequences | | | 50000 | |  | | | |
|  | Blast E value | | | 30000 | |  | | | |
|  | Blast word size | | | 7 | |  | | | |
|  | Max candidate primer pairs | | | 500 | |  | | | |
|  | Min PCR product size | | | 61 | |  | | | |
|  | Max PCR product size | | | 1000 | |  | | | |
|  | Min Primer size | | | 15 | |  | | | |
|  | Opt Primer size | | | 20 | |  | | | |
|  | Max Primer size | | | 25 | |  | | | |
|  | Min Tm | | | 57 | |  | | | |
|  | Opt Tm | | | 60 | |  | | | |
|  | Max Tm | | | 63 | |  | | | |
|  | Max Tm difference | | | 3 | |  | | | |
|  | Repeat filter | | | AUTO | |  | | | |
|  | Low complexity filter | | | Yes | |  | | | |
