# Supplementary material for: A Comprehensive Quality Evaluation System for Complex Herbal Medicine Using PacBio Sequencing, PCR-Denaturing Gradient Gel Electrophoresis, and Several Chemical Approaches
Source: Front Plant Sci. 2017 Sep 13;8:1578. doi: 10.3389/fpls.2017.01578 (PMC5601397; doi:10.3389/fpls.2017.01578)
Supplement: TABLE S4 — Determination results of contaminants. [file Table_4.DOC]

Supplementary Table S4. Determination results of contaminants residue.

| Contamination residues | | Unit | Huangqi (HQ) | | | | Danggui (DG) | | | |
| --- | --- | --- | --- | --- | --- | --- | --- | --- | --- | --- |
|  |  |  | Batch 1 | Batch 2 | Batch 3 | CL | Batch 1 | Batch 2 | Batch 3 | CL^*^ |
| Sulfur dioxide residue | | mg/kg | Neg. | Neg. | Neg. | ≤150 | Neg. | Neg. | Neg. | ≤150 |
| Heavy metal residue | Lead | mg/kg | 0.2 | 0.3 | 0.4 | ≤5 | 0.6 | 1.1 | 0.9 | ≤10 |
|  | Cadmium | mg/kg | 0.01 | 0.03 | 0.03 | ≤0.3 | 0.03 | 0.04 | 0.04 | ≤1 |
|  | Arsenic | mg/kg | 0.2 | 0.2 | 0.2 | ≤2 | 0.5 | 0.6 | 0.4 | ≤5 |
|  | Mercury | mg/kg | Neg. | Neg. | Neg. | ≤0.2 | Neg. | Neg. | 0.005 | ≤1 |
|  | Copper | mg/kg | 4.2 | 5.3 | 5 | ≤20 | 7.4 | 9.4 | 7.6 | ≤20 |
| Pesticide residue | Dichlorodiphenyltrichloroethane | mg/kg | Neg. | Neg. | Neg. | ≤0.2 | Neg. | Neg. | 0.0013 | ≤0.2 |
|  | Hexachlorocyolohexane | mg/kg | Neg. | 0.0016 | Neg. | ≤0.2 | Neg. | 0.0013 | Neg. | ≤0.2 |
|  | Benzene | mg/kg | Neg. | Neg. | Neg. | ≤0.1 | Neg. | Neg. | Neg. | ≤0.1 |
|  | Aldrin | mg/kg | Neg. | Neg. | Neg. | ≤0.02 | Neg. | Neg. | Neg. | ≤0.02 |
| Aflatoxins residue | B1 | μg/kg | Neg. | Neg. | Neg. |  | Neg. | Neg. | 0.67 |  |
|  | Total of B1, B2, G1 and G2 | μg/kg | Neg. | Neg. | Neg. | ≤5 | Neg. | Neg. | 0.67 | ≤5 |
| Harmful microbes’ margin | Aerobic bacteria | cfu/g | ＜10 | ＜10 | ＜10 | ≤10^4^ | ＜10 | ＜10 | ＜10 | ≤10^4^ |
|  | Mold and yeast | cfu/g | ＜10 | ＜10 | ＜10 | ≤10^2^ | ＜10 | ＜10 | ＜10 | ≤10^2^ |
|  | *Escherichia coli* | cfu/g | Neg. | Neg. | Neg. | 0 | Neg. | Neg. | Neg. | 0 |
|  | *Salmonella* | cfu/10g | Neg. | Neg. | Neg. | 0 | Neg. | Neg. | Neg. | 0 |
|  | Bile salt resistant gram-negative bacteria | cfu/g | ＜10 | ＜10 | ＜10 | ≤10^2^ | ＜10 | ＜10 | ＜10 | ≤10^2^ |

*: CL: Control limitation, which is complied with *Green Standards of Medicinal Plants and Preparations for Foreign Trade and Economy* and adjusted according to the *Chinese Pharmacopoeia* (2015 version).
